# A premixed calcium hydroxylapatite/carboxymethylcellulose–hyaluronic acid hybrid filler for skin quality

**DOI:** 10.1016/j.jpra.2026.01.041

**Published:** 2026-01-30

**Authors:** Andrea Lazzarotto, Stefania Guida, Jonathan Kadouch, Nabil Fakih-Gomez, Nicola Zerbinati, Luigi Colombo, Massimo Vitale, Mariana César Corrêa, Kyu-Ho Yi

**Affiliations:** a Department of Maxillofacial and Plastic Surgery, University Hospital “ASST-Lariana” of Como, and Private Practice in Como, Milan and Udine, Italy; b School of Medicine, Dermatology Clinic, Vita-Salute San Raffaele University, IRCCS San Raffaele Scientific Institute, Milan, Italy; c Practice for Aesthetic Dermatology, ReSculpt Clinic, Amsterdam, The Netherlands; d Department of Facial Plastic and Cranio-Maxillo-Facial Surgery, Fakih Hospital, Khaizaran, Lebanon; e Department of Medicine and Surgery, University of Insubria, Varese, Italy; f Private Practice, Bologna, Italy; g Clinica Dermatologica Dra. Mariana Corrêa, São Paulo, Brazil; h Division of Anatomy and Developmental Biology, Department of Oral Biology, Human Identification Research Institute, BK21 FOUR Project, Yonsei University College of Dentistry, Seoul, Republic of Korea

**Keywords:** Calcium hydroxylapatite, Hyaluronic acid, Skin quality, Hybrid filler

## Abstract

**Background:**

Hybrid injectable approaches combining calcium hydroxylapatite/carboxymethylcellulose with hyaluronic acid gels may improve both facial contour and skin quality.

**Methods:**

We retrospectively reviewed 12 women treated with a premixed hybrid filler injected in the subdermal plane across the mid- and lower-face. Outcomes were assessed at baseline and 4 months using the Merz Aesthetic Scales for mid- and lower-face aging severity, the Lemperle Wrinkle Severity Scale (cheek wrinkles), and a 5-point Global Aesthetic Improvement Scale for patient-reported “skin glow.”

**Results:**

All Merz domains and cheek wrinkle severity improved at 4 months (cheek wrinkles, *p* < 0.001). Seven of 12 participants rated their skin glow as “much improved” or “very much improved,” and all 12 reported at least “improved.” No serious adverse events occurred; bruising resolved spontaneously within 1–2 weeks.

**Conclusions:**

In this retrospective case series, the premixed hybrid filler was well tolerated and was associated with improvement in mid- and lower-face aging severity and patient-perceived skin glow at 4 months.

**Level of evidence:**

IV.

## Introduction

Skin quality plays a crucial role in human attractiveness in modern society. It significantly influences the perception of age, beauty, health, and youthfulness. As we age, the skin undergoes notable changes.^1^ High-quality skin is often associated with vitality and youth, serving as the visible interface with one’s social environment.^2^ Various alterations in skin quality are commonly observed as part of the aging process.

Skin aging occurs due to two primary mechanisms: intrinsic aging (chrono-aging), which is time-dependent, and extrinsic aging (photo-aging), caused mainly by environmental factors such as ultraviolet (UV) radiation. This aging process affects all layers of the skin. At the dermal level, notable changes occur in the extracellular matrix (ECM), particularly in collagen and elastin fibers, as well as glycosaminoglycans, including hyaluronic acid (HA). Skin aging is characterized by a reduced synthesis and increased degradation of HA and collagen fibers, leading to decreased skin hydration and a decline in ECM content. In particular, the fragmentation and reduction of collagen fibers diminish the mechanical tension on fibroblasts, thereby reducing their capacity to produce new collagen. As a result, skin elasticity decreases with age, while surface roughness increases, likely due to lower water and sebum content. These changes contribute to the formation of wrinkles and fine lines.^3^

In recent years, minimally invasive procedures such as injectable fillers have revolutionized the landscape of skin rejuvenation, driven by a growing global demand for aesthetic treatments.

To meet patients’ increasing desire for enhanced skin quality and a natural, youthful appearance, attention has turned toward bioactive agents that improve skin turgor and hydration while stimulating collagen production. One such innovation is the use of “hybrid” fillers, combining HA and calcium hydroxylapatite (CaHa).^4^

This study aims to evaluate the effectiveness and safety of a novel hybrid filler composed of cohesive polydensified matrix cross-linked sodium hyaluronate 20 mg/mL and glycerol 17.5 mg/mL (CPM-HA20G), combined with a calcium hydroxyapatite (CaHA) and carboxymethylcellulose (CMC) formulation, for the treatment of skin aging signs and the enhancement of overall facial skin quality.^5^^,^^6^

## Materials and methods

### Study population

Adults (≥18 years) seeking improvement in mid- and lower-face skin quality and/or contour were eligible if they had a baseline Merz Aesthetic Scale (MAS) score of ≥1 (mild to very severe) in at least one relevant domain (upper cheek fullness, nasolabial fold at rest, marionette lines at rest, oral commissure at rest, or jawline contour at rest). Participants were required to return for standardized photography at baseline and Month 4 and to refrain from additional aesthetic procedures in the treated areas during follow-up. Exclusion criteria included hypersensitivity to local anesthetics, connective tissue disease, any facial rejuvenation treatment within the preceding 6 months, prior mid- or lower-face lifting surgery, pregnancy or lactation, systemic immunosuppressant use, severe uncontrolled systemic illness, active inflammatory dermatoses at the injection sites, and prior permanent or semi-permanent fillers in the treatment area. Screening was performed using the official photonumeric MAS atlases; prior to data collection, the primary rater completed a calibration exercise with a senior reviewer using a set of 20 standardized images, and discrepancies were resolved by consensus.

### Hybrid filler composition and preparation

**Figure 1:**
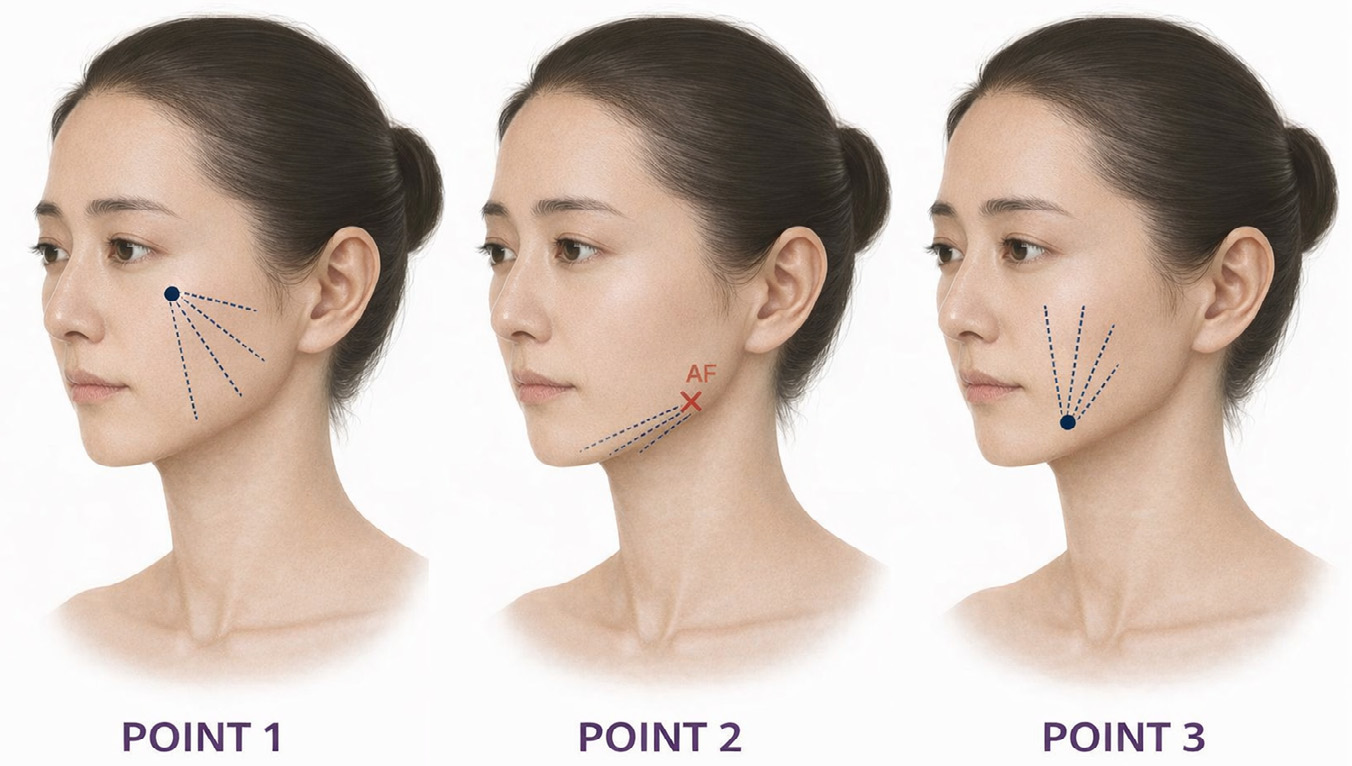
Preparation of the mixture. A 1.5 mL syringe of CaHA was diluted with 0.5 mL of 1% lidocaine and 1 mL of CPM-HA20G, resulting in a total volume of 3 mL.

All treatments were performed by a single investigator (A.L.) in a private practice setting. A premixed hybrid filler was prepared immediately before injection by combining 1.5 mL of a calcium hydroxylapatite/carboxymethylcellulose filler with 1.0 mL of cohesive polydensified matrix hyaluronic acid gel (CPM-HA20G) and 0.5 mL of 1% lidocaine (total volume, 3.0 mL), yielding a 1:1 dilution of CaHA/CMC with the diluent (CPM-HA20G + lidocaine) by volume. A 1:1 dilution was selected to balance subdermal spreadability for skin-quality improvement while maintaining sufficient cohesivity to allow controlled cannula delivery and mid-/lower-face support; alternative dilution ratios (e.g., 1:2 or 1:3) may yield different clinical effects and warrant comparative evaluation. The components were homogenized using a transfer adapter by transferring the mixture between two Luer-lock syringes for a minimum of 20 complete passes, followed by two standardized aspiration–expulsion (“foaming”) cycles (each cycle defined as one slow aspiration and one expulsion through the adapter), and then an additional 10 passes to re-homogenize the suspension. The final mixture was used immediately after preparation. (Figure 1). For reproducibility, the preparation was considered complete only after the above minimum sequence (≥20 passes, 2 foaming cycles, and 10 final passes) was achieved for every case. Rheological characterization of the premixed suspension was not performed in this retrospective series; therefore, consistency was supported by standardized component volumes and a fixed minimum mixing/foaming sequence.

### Injection technique

**Figure 2:**
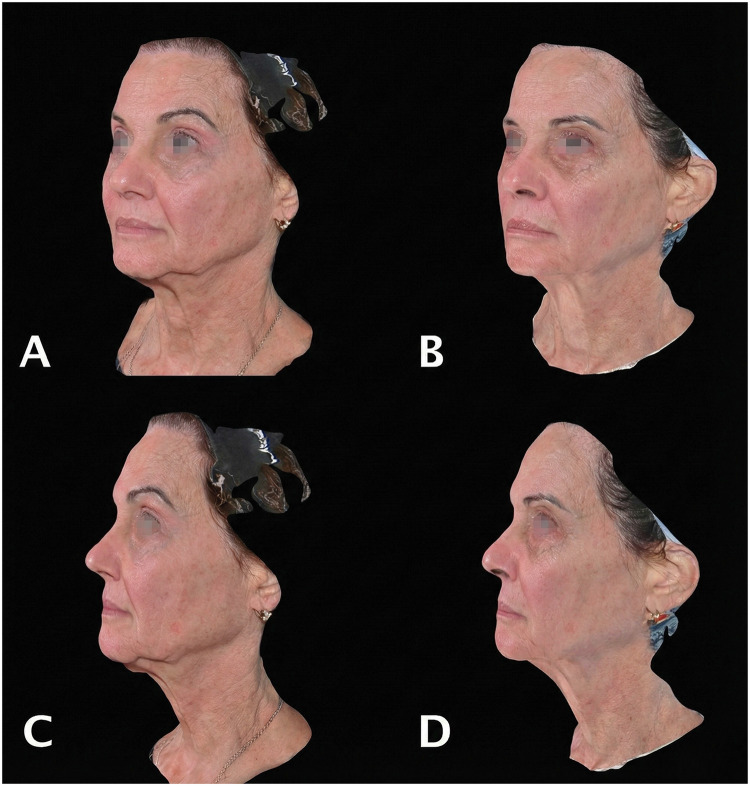
The 4-point injection pattern. Treatment areas and volume of material used per injection site with retrograde fanning vectors.

The skin was cleansed with chlorhexidine, and a thin layer of 3% lidocaine topical anesthetic cream was applied for approximately 30 min and removed before injection. For each hemi-face, four entry points were marked: (1) at the level of the zygomatic arch (1 cm anterior to the zygomatic ligament), (2) at the mandibular angle (1.5 cm anterior to the gonial angle), (3) at the level of the modiolus (1 cm lateral to the oral commissure), and (4) at the pre-jowl sulcus (1 cm anterior to the sulcus) (Figure 2).

Local infiltration anesthesia was administered at each entry point with 1% lidocaine with epinephrine 1:100,000. Because this was a retrospective review, exact topical and infiltration lidocaine amounts were not consistently quantifiable across charts; this is a limitation and should be prospectively standardized and reported in future studies. The amount of lidocaine added directly to the filler was fixed (0.5 mL of 1% lidocaine, equivalent to 5 mg) and therefore unlikely to contribute meaningfully to systemic toxicity. The hybrid filler was delivered in the subdermal plane using a 25-G, 50-mm blunt-tip cannula (STERiGLIDE®, TSK Laboratory, Tochigi-shi, Japan) with retrograde fanning; approximately 0.1 mL was deposited per pass (Table 1).

**Table 1:** Recommendation for injection.

Areas	Entry point	Volume	Injection plane	Technique
Malar region	Zygomaticofrontal suture	0.3 mL	Subcutaneous	Fanning 3 lines
Cheek region	Mandibular angle	0.5 mL	Subcutaneous	Fanning 5 lines
Pre-jaw region	Pre-jowl sulcus	0.4 mL	Subcutaneous	Fanning 4 lines
Nasolabial region	Modiolus	0.3 mL	Subcutaneous	Fanning 3 lines

### Assessment

Standardized two-dimensional photographs were obtained at baseline (T0) and Month 4 (T1) in five views (frontal, right and left lateral, and right and left 45° oblique) using a Nikon D3 digital SLR camera (Nikon Corporation, Tokyo, Japan) with consistent lighting and positioning. An independent evaluator (not involved in treatment) assessed outcomes from photographs.

Clinical efficacy was evaluated using the MAS for upper cheek fullness, nasolabial fold at rest, marionette lines at rest, oral commissure at rest, and jawline contour at rest. Each MAS is a 5-point photonumeric scale ranging from 0 (none) to 4 (very severe). Cheek wrinkle severity was assessed using the Lemperle Wrinkle Severity Scale (0–5). At Month 4, patients rated the perceived “skin glow” improvement using the 5-point Global Aesthetic Improvement Scale (GAIS) (worse, no change, improved, much improved, very much improved).

### Safety

Medical history and a focused examination of the treatment area were performed before injection. Pain was recorded on a 0-10 numerical rating scale at two time points: immediately prior to cannula insertion after topical anesthetic removal and 2-3 min after local infiltration anesthesia at each entry point. Patients were observed for 30 min post-procedure for immediate reactions (erythema, oedema, ecchymosis). Adverse events were recorded throughout follow-up and classified as minor (no medical intervention) or major (requiring medical intervention).

### Statistical analysis

Given the retrospective case-series design and small sample, analyses were exploratory. The primary efficacy outcome was the paired change in the Lemperle cheek-wrinkle score from baseline (T0) to Month 4 (T1), where a reduction of ≥1 grade was considered clinically meaningful. Secondary outcomes were paired changes in the five MAS domains and GAIS categories. Because outcomes were ordinal and repeated within subjects, paired changes from T0 to T1 were analyzed using the Wilcoxon signed-rank test (two-sided α = 0.05). Multiplicity across the five MAS tests was controlled using the Benjamini–Hochberg false discovery rate (q = 0.05). Continuous variables are summarized as mean ± standard deviation or median (interquartile range), as appropriate. Categorical outcomes are presented as counts (n/N) given the small sample size. To explore potential confounding by baseline severity, we additionally assessed the association between baseline domain scores and magnitude of change at Month 4 using simple regression (change score as outcome; baseline score as predictor) as a sensitivity analysis. Analyses were performed in SPSS Statistics v28 (IBM Corp., Armonk, NY, USA) and cross-checked in R v4.3.0 (R Foundation for Statistical Computing, Vienna, Austria).

This study is reported in accordance with the STROBE guidelines.

## Results

**Figure 3:**
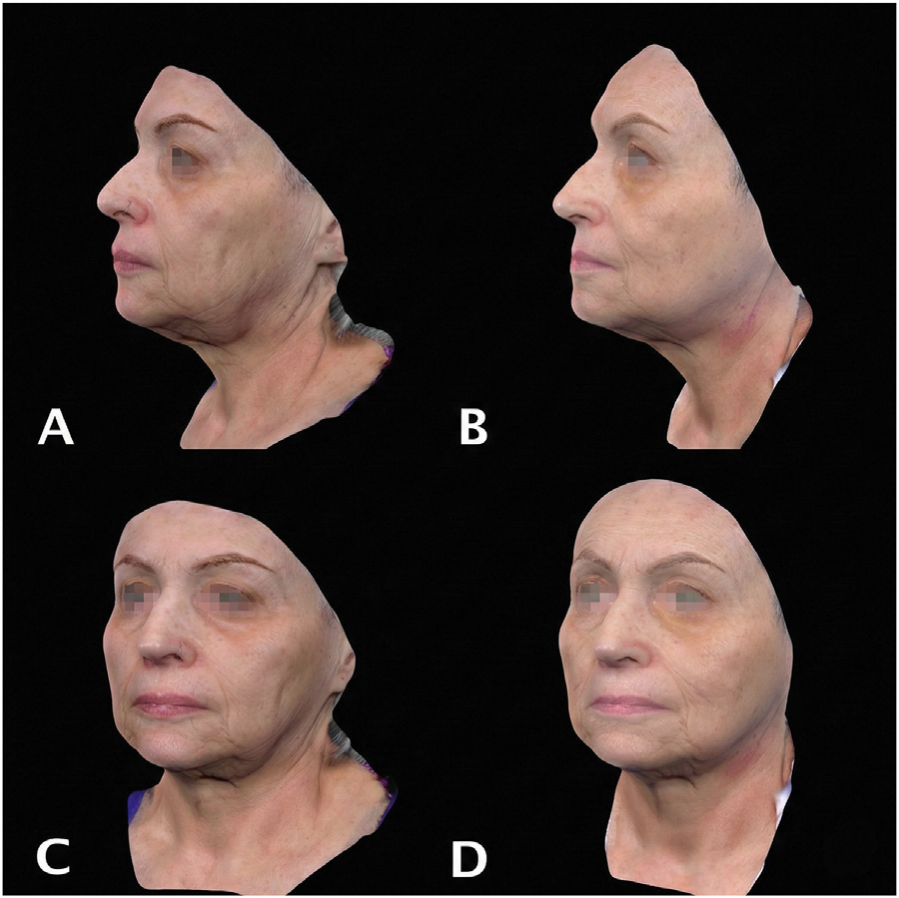
A 44-year-old female underwent treatment with a premixed hybrid filler injection (1.5 mL of CaHA), 1 mL of CPM-HA20G, and 0.5 mL of 1% lidocaine). Frontal, right three-quarter, and lateral views are shown. A) Before treatment. B) Baseline. C) Four-month follow-up.

Twelve women were included (mean age 56.7 ± 7.5 years; range 36-68). Baseline severity scores by domain are summarized in Table 2. At 4 months (T1), all assessed mid- and lower-face Merz Aesthetic Scale domains (upper cheek fullness, nasolabial folds at rest, marionette lines at rest, oral commissures at rest, and jawline contour at rest) improved compared with baseline (T0) (Table 3; Figure 3).

**Table 2:** Variations in skin aging severity scales before treatment (T0) and at 4-month follow-up (T1) in the study population.

Areas	T0, mean ± SD	T1, mean ± SD	*p*-value
Upper cheek fullness	3.7 ± 0.9	2.8 ± 0.7	<0.001
Nasolabial fold at rest	3.9 ± 0.8	2.2 ± 0.6	<0.002
Marionette lines at rest	3.3 ± 1	2 ± 0.7	<0.003
Oral commissures at rest	3.1 ± 0.8	2 ± 0.9	<0.004
Jawline at rest	3.6 ± 1	2 ± 0.8	<0.005

**Table 3:** Distribution of wrinkle severity scores according to the Lemperle Scale.

T0, mean ± SD	T1, mean ± SD	*p*-value
3.6 ± 0.9	2.2 ± 1.0	0.000

Cheek wrinkle severity also decreased, with the mean Lemperle score improving from 3.6 ± 0.9 at T0 to 2.2 ± 1.0 at T1 (Table 3; *p* < 0.001).

For patient-reported “skin glow” at T1, 7/12 participants rated the outcome as “much improved” or “very much improved,” while 5/12 rated it as “improved” (Table 4).

**Table 4:** Distribution of skin radiance outcomes based on the patients’ Global Aesthetic Improvement Scale (GAIS).

GAIS	*N* (%)
Very much improved	4 (33)
Much improved	3 (22)
Improved	5 (45)
No improvement	0
Worse	0

All patients completed treatment without serious AEs or signs of vascular compromise. Injection-phase pain was low: pre-infiltration NRS median 3 (IQR 2–4), post-infiltration NRS median 1 (IQR 0–2); overall session NRS mean 2.1 ± 1.0, median 2 (IQR 1–3). Immediate, self-limited reactions included erythema (9/12), edema (8/12), and ecchymosis/bruising (7/12), resolving in a median of 6 days (IQR 4–8). No vasovagal events, nodules, Tyndall effect, delayed hypersensitivity, or infections were observed through Month 4. No patient required rescue analgesia.

## Discussion

This study presents the clinical outcomes of a novel hybrid filler treatment combining CaHA/CMC and CPM-HA20G, evaluating its efficacy in improving facial skin aging and overall skin quality, as well as its safety profile. While both products have been traditionally used together in facial aesthetic procedures, they have not previously been administered as a pre-mixed formulation.^7^

CaHA was injected into the subcutaneous layer to provide volumization and structural support through collagen stimulation. CPM-HA20G was included to enhance skin revitalization, targeting improvements in hydration, elasticity, and texture.^6^ Previous studies have investigated the use of hybrid fillers combining CaHA/CMC with various HA formulations, primarily focusing on lifting and contouring effects. However, limited data are available regarding their impact on skin aging and quality. Furthermore, existing research has largely explored diluted or hyperdiluted CaHA using various ratios, without assessing the specific combination of CaHA/CMC and CPM-HA20G in a single injectable suspension for facial application.^8^

Our study aimed to address this gap by examining the synergistic regenerative potential of CaHA/CMC and CPM-HA20G. CaHA stimulates collagen production, while CPM-HA20G contributes to hydration and viscoelastic enhancement. The results demonstrated significant improvements in facial aging signs, as shown by improvements in validated severity scales across mid and lower facial areas. Additionally, wrinkle severity and patient-reported “glowing” effects also showed notable enhancement, indicating improved skin quality.

These effects can be attributed to the biological actions of the filler components. CaHA promotes neocollagenesis, while the cross-linked HA and glycerol in CPM-HA20G enhance hydration and improve the skin’s mechanical properties. The glycerol component may also contribute to prolonged moisture retention and possibly support collagen stabilization, as suggested by in vitro data.^6^

The safety profile of the treatment was favorable. All adverse events were minor, with the most common being transient injection-site hematomas, which resolved spontaneously within a few days. No serious or long-term complications were observed, and the procedure was well-tolerated by all patients.

Our findings regarding improvements in skin aging align with previous studies using diluted or hyperdiluted CaHA (e.g., 1:1 or 1:2 dilution with saline or lidocaine) for facial rejuvenation.^9^ Importantly, this study lacked a control or comparator arm (e.g., CaHA alone, HA alone, or sham), and outcomes were assessed over a short follow-up. Accordingly, natural temporal changes, regression to the mean, and placebo effects cannot be excluded, and causality cannot be definitively attributed to the intervention. Comparative prospective studies are needed to quantify any incremental benefit of the premixed hybrid approach over established monotherapies. However, improvements in wrinkle severity and skin glow have not been previously reported, likely due to the absence of CPM-HA20G in those protocols. In fact, prior reports describe the use of this hybrid filler composition (CaHA/CMC and CPM-HA20G) only for hand rejuvenation treatments.

Moreover, most existing literature on hybrid fillers has focused on contouring and lifting effects rather than skin quality enhancement. These studies also typically used higher filler volumes (mean of 5.1 mL) and often required reinjection within 3 months to maintain results.^10^

The combination of CaHA/CMC and CPM-HA20G as a hybrid filler appears to be a safe and effective approach for facial rejuvenation. This formulation not only improves the appearance of skin aging in the mid and lower face but also enhances skin quality, particularly in terms of wrinkle reduction and the perception of a glowing complexion. These results were achieved in a single treatment session with a relatively low product volume, suggesting a cost-effective and efficient strategy for minimally invasive skin rejuvenation.

Despite these promising findings, the study has several limitations. All injections were performed by a single operator, which limits assessment of inter-practitioner reproducibility and generalizability across different clinical settings. The small sample size (*n* = 12) and retrospective design limit statistical power and generalizability; therefore, findings should be interpreted as exploratory and hypothesis-generating rather than confirmatory. Furthermore, we did not assess the rheological properties of the premixed hybrid suspension; therefore, batch-to-batch mechanical consistency cannot be objectively confirmed and should be investigated in future studies. Future studies are warranted to explore the applicability of this protocol to other anatomical areas affected by skin aging, such as the neck and décolletage, to further expand its clinical utility.

## Conclusion

In this retrospective case series, a premixed calcium hydroxylapatite/carboxymethylcellulose and cohesive polydensified matrix hyaluronic acid gel was well tolerated and was associated with improvement in mid- and lower-face aging severity and cheek wrinkle scores at 4 months, with patient-reported improvement in skin glow. Prospective comparative studies with larger and more diverse cohorts, longer follow-up, and objective skin biophysical measurements are warranted.

## Role of funding source

This research received no specific grant from any funding agency in the public, commercial, or not-for-profit sectors. The funders had no role in study design; data collection, analysis, or interpretation; manuscript preparation; or the decision to submit for publication.

## Informed consent

Written consent for the procedure was obtained as part of routine care.

## Author contributions

Writing-original draft preparation: Andrea Lazzarotto, Stefania Guida, Jonathan Kadouch. Writing-review and editing: Andrea Lazzarotto, Nabil Fakih-Gomez, Nicola Zerbinati. Visualization: Luigi Colombo, Massimo Vitale, Mariana César Corrêa. Supervision: Kyu-Ho Yi.

## Declaration of competing interest

A.L., S.G., N.FG., and J.K. are consultants for Merz Aesthetics (Frankfurt, Germany). A.L. is also a consultant for Fidia Pharma. The other authors declare no conflicts of interest.
